# Correlation between physical activity and adolescent idiopathic scoliosis: a systematic review

**DOI:** 10.1186/s12891-023-07114-1

**Published:** 2023-12-19

**Authors:** Xiang Qi, Chao Peng, Pinting Fu, Aiyuan Zhu, Wei Jiao

**Affiliations:** 1 School of Sport Medicine and Rehabilitation, Beijing Sport University, Beijing, China; 2 School of Physical Education, Taizhou University, Taizhou, China; 3 Department of Rehabilitation, The First Affliated Hospital, Jiangxi Medical College, Nanchang University, Nanchang, China; 4 Department of Rehabilitation, Shanxi Acupuncture Hospital, Taiyuan, China

**Keywords:** Activity, Exercise, Review, Scoliosis

## Abstract

**Background:**

The multifactorial aetiology of scoliosis is well known. Physical activity is considered both a treatment and causative factor for idiopathic scoliosis; however, evidence for a causal relationship between physical activity levels and idiopathic scoliosis in adolescents is conflicting. Therefore, we aimed to summarise the current evidence regarding the association between adolescent idiopathic scoliosis and physical activity and further to assess whether the relationship is dose dependent.

**Methods:**

PubMed, Cochrane, Scopus, and Web of Science databases were searched from 1991 to July 2022 using the following main keywords: adolescent idiopathic scoliosis, physical activity, and risk factors, supplemented with manual searches, secondary citations, and reference searches. The quality of the included literature was evaluated using the Scale for Reporting Observational Studies in Enhanced Epidemiology guidelines.

**Results:**

Eight studies were included in this review, of which six reported an association between adolescent idiopathic scoliosis and physical activity levels and two reported no association. One British study reported reduced physical function early in life as a new risk factor for scoliosis onset.

**Conclusions:**

Physical activity is strongly associated with adolescent idiopathic scoliosis. Physical activity should be encouraged as it plays an important role in the prevention of adolescent idiopathic scoliosis. Further research is needed to determine the dose-dependent relationship between physical activity and prevention of adolescent idiopathic scoliosis.

**Supplementary Information:**

The online version contains supplementary material available at 10.1186/s12891-023-07114-1.

## Background

Physical activity is defined as any bodily movement produced by skeletal muscles that requires energy expenditure [1]. The 2020 World Health Organization (WHO) guidelines call for children and adolescents to undertake at least 60 min per day of moderate-to-vigorous-intensity, mostly aerobic, physical activity; they also recommend that vigorous-intensity aerobic activities and those that strengthen the muscles and bones should be incorporated at least three days per week [2]. However, a global study found that 81% of students aged 11–17 years are physically inactive [3]. A lack of physical activity poses a great threat to the health of children and adolescents. Physical activity is negatively associated with mental illnesses, such as depression, stress, negative affect, and overall physical distress, while positively associated with mental health, such as self-image, satisfaction with life and well-being, and psychological well-being [4–6].

Adolescent idiopathic scoliosis (AIS) is a spinal deformity that occurs in adolescence and is characterised by manifestations such as spinal rotation and scoliosis [7]. AIS often occurs in adolescent women, and the incidence rate in adolescent females is approximately twice as high as that in males [8]. The disease affects the appearance and body shape of patients, induces low back pain, and, in patients with severe scoliosis, decreases cardiopulmonary function, thereby seriously affecting the physical and mental health and quality of life of patients [9–11].

At present, the cause of AIS is unclear and may be related to many factors, such as genetics, environment, hormones, metabolism, and neurology [12]. Several studies had found that patients with scoliosis have common characteristics of taller stature, low systemic bone mass, and lower body mass index [13]. A study showed that patients with AIS have poorer bone mineral density, which is associated with curve severity and is more likely to persist beyond the peripubertal period and achievement of peak bone mass compared to healthy controls [14]. Moreover, several studies have demonstrated abnormalities related to posture, proprioception, and equilibrium control in patients with AIS [15–17]. Exercise has been shown to improve bone mineral density, balance, vestibular function, cognition, and executive abilities in adolescents [2, 18]. Physical activity plays an important role in the growth and development of adolescents, with AIS occurring during this period. However, the relationship between physical activity and AIS development remains unclear; previous studies have yielded conflicting outcomes, with some suggesting no association [19, 20], some reporting higher physical activity levels in patients with scoliosis [21, 22], and others finding lower physical activity levels in patients with scoliosis [23, 24]. The different associations seen may reflect differences in the validity and reliability of the methods used to measure physical activity levels, and some studies have relied on the memory of guardians or adolescents to complete the collection of information on activity levels, which reduces the accuracy of information collection. Studies with different outcomes used different populations, and exercise programs lacked homogeneity. Other studies did not take into account the effect of modifying factors or confounding factors.

Therefore, this review aimed to synthesise the current evidence and summarise the association between physical activity and the incidence of AIS. We also aimed to determine whether this relationship follows a dose-dependent pattern, thereby providing a scientific basis for preventing AIS.

## Methods

This review was registered with PROSPERO (registration number: CRD42022309032). The Technical Expert Group was made up of five physiatrists with experience in idiopathic scoliosis (W.J., X.Q., C.P., PT.F., AY.Z.). Two reviewers (PT.F. and AY.Z.) independently reviewed qualifications and abstracts of eligible studies, and the quality of the included studies was independently evaluated by two researchers (X.Q. and C.P.). Disagreements were resolved through discussion. According to the quantitative scoring method of the STROBE checklist prepared by Limaye et al., publications were classified as excellent, good, fair, or poor based on their overall scores [25].

### Search procedure

A computerised search was conducted for original peer-reviewed research journal articles published in English before July 2022 in the PubMed, Cochrane, Scopus, and Web of Science databases. The keywords of the search were as follows: (“Physical Activity” [All Fields] OR “Exercises” [All Fields] OR “Physical Exercise” [All Fields] OR “Acute Exercise” [All Fields] OR “Isometric Exercise” [All Fields] OR “Aerobic Exercise” [All Fields] OR “Exercise Training” [All Fields]) AND (“scoliosis” [All Fields] OR “spinal curvature” [All Fields] OR “AIS” [All Fields] OR “Adolescent Idiopathic Scoliosis” [All Fields]).

### Inclusion criteria

We included English articles with cross-sectional, case-control, or cohort study designs. The participants included, but were not limited to, patients aged 6–18 years with AIS confirmed by clinical diagnosis and laboratory examination. There were no restrictions imposed regarding race, nationality, and disease course. Data on physical activity and the association between different types of exercise and AIS were extracted. The outcome index included articles on AIS incidence.

### Exclusion criteria

We excluded studies with incomplete or poorly described data and those with no specific clinical outcomes. We also omitted any articles for which complete details were not available in the abstract alone.

## Results

**Fig. 1:**
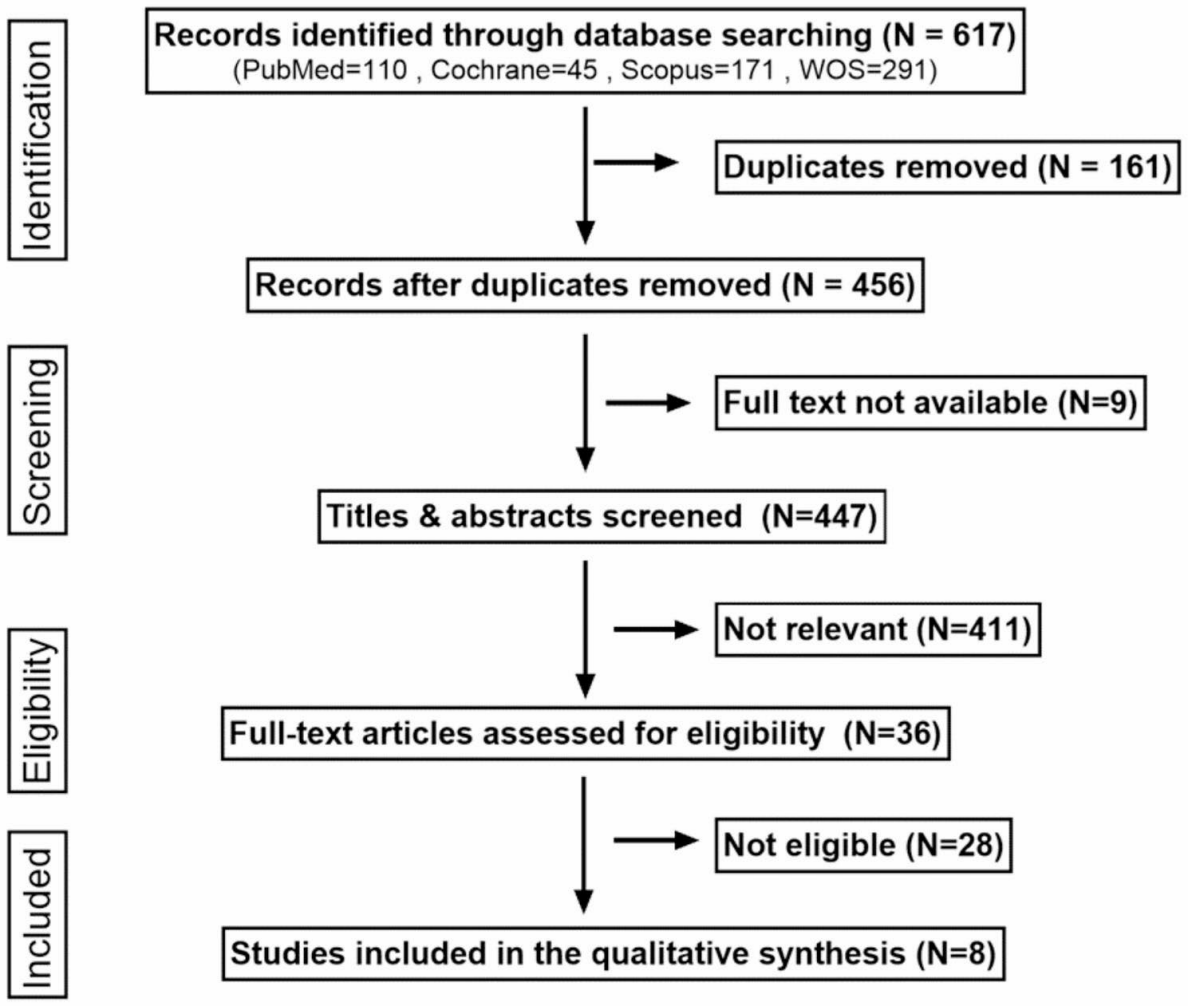
An expanded PRISMA flow chart showing the selection process of studies included in the systematic review

After eligibility assessment, eight articles were included in this systematic review (Fig. 1). Five of the articles were cross-sectional studies [19, 26–29], two were case-control studies [30, 31], and one was a cohort study [32]. Table 1 summarises the main findings of the quality assessment (with a full description in Supplementary Appendix S1).

**Table 1:** Quality assessment of the studies included in this literature review

Study	Methods										Results				Discussion	
	Objectives and rationale	Study design	Setting	Participants’ eligibility matching criteria	Variables	Data sources and measurements	Addressing potential sources of bias	Sample size	Quantitative variables	Statistical methods	Participants	Descriptive data	Outcome data	Main results and other analyses	Generalizability	Funding and conflicts of interest
Kenanidis et al.(2008)	L	H	L	H	L	H	L	L	L	L	L	H	L	H	H	L
Tobias et al.(2019)	L	L	L	H	L	H	L	H	L	L	H	H	L	L	L	L
Golalizadeh et al.(2020)	L	H	L	L	L	H	H	L	L	L	L	H	H	H	H	L
Assis et al.(2021)	L	L	H	L	L	H	L	L	L	H	H	H	L	L	H	L
Cai et al.(2021)	L	L	L	H	L	H	L	L	L	L	H	L	L	L	L	L
Watanable et al.(2017)	L	H	L	L	L	H	H	H	L	L	L	L	L	L	L	L
Mcmaster et al.(2015)	L	L	H	H	L	H	H	H	L	H	L	L	H	L	L	L
Scaturro et al.(2021)	L	H	L	L	L	H	H	H	L	L	H	L	H	H	H	L

H, high risk of bias; L, low risk of bias

The characteristics of the studies included in this review are summarised in Table 2. The total number of participants in the eight studies was 13,473. The AIS diagnosis was confirmed by imaging and movement testing in 3,602 (27%) patients. Among these studies, five described the sex of patients [19, 27, 28, 30, 31], and the prevalence of AIS was higher in girls than in boys. Four studies were conducted in European countries, including Greece, Scotland, the UK, and Switzerland [19, 29, 31, 32]; three in Asian countries, including Japan, Iran, and China [26–28]; and one study was conducted in South America (Brazil) [30]. One study focused on athletic populations [19]. Two studies measured physical activity using the International Physical Activity Questionnaire [26, 30]. One study used the Avon Longitudinal Study of Parents and Children to collect self-reported physical fitness/activity data at 18 months and 10 years of age and used an accelerometer to obtain physical activity data of 11-year-old children over 1 week [32]. Six studies concluded that physical activity levels are associated with the onset of adolescent scoliosis in a dose-response manner [27–32]. Cai et al. [27] found that lower levels and shorter durations of physical activity [odds ratio (OR): 7.29, 95% confidence interval (CI): 1.99–53.37] were significantly associated with adolescent scoliosis. With an increase in exercise time, the incidence of scoliosis gradually decreased, and individuals who exercised < 1 h per day were 7.29 times more likely to develop scoliosis than those who exercised 3 h per day. Watanabe et al. [28] found that adolescent scoliosis was associated with classical ballet training, and the odds of AIS increased with an increase in a child’s training frequency, years of experience, and duration of ballet training. Scaturro et al. [29] found that engaging in high-risk sports (dance or artistic gymnastics) or physical activity for < 3 h per week was significantly associated with adolescent scoliosis and suggested that the number of hours per week spent on sports may affect the risk of AIS. Tobias et al. [32] found that a higher quartile of moderate and vigorous physical activity was associated with a lower risk of scoliosis.

**Table 2:** The characteristics of included studies

Authors	Article title	Age	N=	Country in which study was conducted	AIS Patients	Diagnostic criteria	Physical Activity	Reported outcome
Kenanidis, E., et al.	Adolescent idiopathic scoliosis and exercising - Is there truly a liaison?	Athletes Mean	N = 2387 (female = 1210;male = 1177)Athletes = 1134 (male = 624 / female = 510); Nonathletes = 1253 (male = 553 / female = 700)	Greece	99 (Athletes = 48:	Imaging assessment of suspected patients	Active, consistent and systematic practice of a sport for at least 2 years and compliance with a minimum of 10 hours of professional training per week prior to participation in the study. / Not practising any sport, or not practising any sport systematically, or practising a sport for recreational purposes only	Systematic exercise may not be associated with the development of AIS. Active physical activity also does not appear to affect the extent of major scoliosis
		(male = 13.32			male = 20 /female = 28;			
		(SD 0.89)			Nonathletes = 51: male = 16 / female = 35)			
		Female = 13.44						
		(SD 0.87)						
		Nonathletes Mean						
		(male = 13.41						
		(SD 0.84)						
		Female = 13.45						
		(SD 0.83)						
Golalizadeh, D., et al.,	Faulty posture: Prevalence and its relationship with Body Mass Index and Physical Activity among female adolescents	Range = 14–18	N = 400	Iran	16	Scoliosis Instrument	Low, Moderate, Severe (International Physical Activity Questionnaire ,IPAQ) Physical activity level in the last week	There was no significant correlation between an individual’s level of physical activity (according to the IPAQ questionnaire) and postural disorders such as shoulder asymmetry, knee inversion, knee valgus and pronation.
Cai, Z., et al.,	Morphology and epidemiological study of idiopathic scoliosis among primary school students in Chaozhou, China	Range = 6–12	N = 2547	China	male = 122 female = 216	Standing observation Adams test	Length of exercise per day:	Lower body weight, myopia, inadequate sleep time and less physical activity were associated with IS. Insufficient sleep (OR = 2.65, 3.33) ), and less time spent exercising (OR = 7.09, 7.29) were significantly associated with IS. The incidence of scoliosis decreased progressively with increasing time spent exercising, with those who exercised less than 1 h per day being 7.29 times more likely to do so than those who exercised 3 h per day.
			(female = 1094				more than 3 hours;	
			male = 1453)				2–3 hours;	
							1–2 hours;	
							less than 1 hour	
Watanabe, K., et al.,	Physical Activities and Lifestyle Factors Related to Adolescent Idiopathic Scoliosis.	Range = 10–14	N = 2759(female)	Japan	2600	Full spine radiographs	Classical ballet training, basketball, badminton	AIS was associated with classical ballet training (OR, 1.38; 95% confidence interval [CI], 1.09 to 1.75); the odds of developing AIS increased as the child’s frequency of training, years of experience and time spent in ballet training increased. Basketball and badminton training were negatively associated with AIS.
							starting age, years of experience, frequency (1 session, 2–3 sessions, 4 sessions per week) duration	
Scaturro, D, et al.,	Risk factors, lifestyle and prevention among adolescents with idiopathic juvenile scoliosis: A cross sectional study in eleven first-grade secondary schools of palermo province, italy.	Range=	N = 428	Switzerland	47	Adams Testing Bunnel tiltmeter	High-risk sports (dance or artistic gymnastics);	Suspected AIS cases were associated with high-risk exercise (*p* < 0.05), physical activity lasting ≥ 3 hours per week (*p* < 0.05), low back pain (*p* < 0.001), postural disturbance (*p* < 0.01) and no contact with a physician (*p* < 0.01). High-risk exercise (adj OR = 1.83; CI 95% 1.11–4.76) and postural disturbance (adj OR = 1.67; CI 95% 1.12–3.60) practices were shown to be significantly associated with a definitive diagnosis of AIS (Cobb angle ≥ 10° X-rays)
		11–14 Mean=					hours of physical activity per week:	
		11.7(SD0.85)					less than or equal to 3 hours;	
							more than 3 hours	
Assis, S.J.C., et al.,	Influence of physical activity and postural habits in schoolchildren with scoliosis.	Range=	N = 156 (female		156	Adams Testing	International Physical Activity Questionnaire (IPAQ)	The correlation between physical activity and ‘scoliosis’ is statistically significant. Low physical activity and the categorisation of schoolchildren as irregularly active are considered risk factors for scoliosis, but postural habits do not appear to be associated with this condition.
		12–17 Mean=	55.1%/	Brazil			Very active;	
		13.9	male44.9%)				Active;	
							Irregularly active;	
							Irregularly active;	
							Sedentary;	
							Review of records for the past 5 years	
McMaster, M.E., A.J. Lee, and R.G. Burwell,	Physical activities of Patients with adolescent idiopathic scoliosis (AIS): preliminary longitudinal case-control study historical evaluation of possible risk factors.	AIS Mean=	N = 156	Scotland	79(female = 66/	A spinal surgeon diagnosed	Participation in dance, gymnastics, karate, swimming, football, hockey and rowing at least once a week after the age of 5	Progressive AIS was positively associated with social deprivation, early introduction to a heated indoor pool and toe touch ability. AIS was negatively associated with participation in dance, skating, gymnastics or karate, football or hockey classes, which may suggest the possibility of prevention.
		15.1 / Control group Mean=			male = 13)			
		14.7						
Tobias, J.H., et al.,	Association between physical activity and scoliosis: a prospective cohort study.	Mean = 15	N = 4640	England	267	Full body supine DXA scan	Self-reported data: 18 months and 10 years activity (average number of times participating in vigorous physical activity in the past month)	Lower physical activity is a new risk factor for the onset of scoliosis.
							Objective data: 7 days physical activity (moderate, vigorous activity, light activity or quiet minutes per day) measured using an activity recorder	

Two case-control studies found that physical activity was significantly associated with scoliosis in adolescents. de Assis et al. [30] found that minimal physical activity was a risk factor for scoliosis (OR: 2.81, 95% CI: 1.04–7.57, *P* = 0.041). Low and irregular physical activities have been identified as risk factors for adolescent scoliosis. McMaster et al. [31] found that children who did not participate in dance (girls only), gymnastics, or karate classes or those who regularly participated in horseback riding and skating had higher rates of AIS than children who participated in these activities. A prospective cohort study identified low physical activity levels as a new risk factor for scoliosis [32]. Tobias et al. [31] found that infants who were able to stand up without support at 18 months of age were 66% less likely to develop scoliosis at 15 years of age than those who were unable to stand up (OR: 0.34, 95% CI: 0.13–0.90, *P* = 0.030). Children whose mothers reported their most vigorous physical activity at the age of 10 years were 53% less likely to have scoliosis (OR: 0.47, 95% CI: 0.24–0.92, *P* = 0.027). Those with more objectively measured moderate/vigorous physical activity at 11 years of age were 30% less likely to have scoliosis (OR: 0.69, 95% CI: 0.59–0.82, *P* < 0.001). This suggests that, as early as 18 months of age, reduced physical ability and activity are associated with an increased risk of scoliosis flares between 10 and 15 years of age.

Two cross-sectional studies concluded that physical activity was not associated with AIS. Kenanidis et al. [19] concluded that systematic physical exercise might not be associated with the development of AIS. Golalizadeh et al. [26] reported no statistically significant relationship between AIS and physical activity levels.

## Discussion

In this review, we summarise the available evidence regarding the association between AIS and physical activity levels. The evidence is inconsistent; two studies showed no statistically significant association between physical activity level and the occurrence of adolescent scoliosis, and six reported a statistically significant association.

In a cross-sectional observational study conducted in a physical education school by Kenanidis et al., the physical activity performed by students was relatively high, and the results of the study concluded that physical activity was not associated with AIS, suggesting that physical activity for the prevention of AIS follows a dose effect [19]. It was determined that higher levels of physical activity are not better than lower levels for the prevention of AIS and that moderate-intensity physical activity is better than other intensities for the prevention of AIS. In a cross-sectional study by Golalizadeh et al., the sample size was too small (n = 16) and the use of the IPAQ questionnaire to measure physical activity in growing adolescents was inaccurate, as adolescent physical activity levels were variable. Moreover, short-term exercise may have little effect on AIS [26], and focus should be placed on the association between long-term exercise and AIS.

Six other studies, including two case-control studies, reported an association between physical activity and AIS. In schoolchildren, low and irregular physical activity were considered risk factors for scoliosis [30]. Regular weekly ball sports training, skating, or karate sessions were found to be negatively associated with AIS [31]. This may be because ball-based activities can improve neuromotor control, as well as trunk and pelvic stability. Studies have also reported positive associations between AIS, early indoor warm-water swimming, and engaging in high-risk sports in early childhood [31]. This observation is consistent with those of other studies that have reported a positive association between forms of physical activity, such as dancing [33], rhythmic gymnastics [34], swimming [35], and others, and the risk of AIS. This may be due to the excessive asymmetric movement of the spine caused by these activities, which increases the probability of excessive joint movement, leading to the deformation and rotation of the spine. A prospective cohort study supported the hypothesis of a relationship between physical activity level and AIS and identified reduced physical function in early life as a novel risk factor for scoliosis initiation, presumably because scoliosis deformities occur due to load reduction [32]. This is consistent with another study, wherein Marinov et al. reported that patients with AIS perform significantly lower levels of physical activity than their healthy peers and that most children with AIS do not meet the WHO minimum level of physical activity (mean PAQ-A score of AIS cases 2.59 < 2.75) [36].

Low physical activity levels are significantly associated with both area and volumetric bone mineral density (BMD) of the spine and hip [24]. Studies have shown that individuals with AIS have a lower BMD than their healthy counterparts [37, 38]. During childhood and adolescence, it is necessary to engage in weight-bearing physical activities, such as running and jumping, to achieve peak bone mass and maintain healthy bone mineral accumulation [39–42].

Li et al. [14] found that lower BMD in patients with AIS was associated with curve severity and may persist beyond puberty and the peak bone mass stage. Hui et al. [43] found that physical activity and exercise participation levels were generally lower in patients with AIS and were associated with lower BMD, skeletal muscle mass, muscle strength, and quality of life. Scoliosis due to inadequate BMD is an important aetiology of AIS and may indicate a correlation between physical inactivity and BMD in patients with AIS. Furthermore, physical activity in an outdoor environment is more likely to promote vitamin D and calcium absorption in adolescents, and it has been documented that calcium and vitamin D supplementation can help in the management of AIS [38, 44]. Therefore, further research is needed to explore the relationship between physical activity and BMD in patients with AIS.

In recent years, several restrictive policies derived from the coronavirus pandemic have led to school and park closures and the cancellation of physical activity classes, which can prevent adolescents from achieving the WHO-recommended level of physical activity; adolescents were forced to stay at home and lacked access to exercise and peers. One study found that short-term changes in the physical activity and sedentary behaviour of children in the United States as a result of the new coronary pneumonia epidemic are permanently ingrained and may lead to an increased risk of childhood obesity, diabetes, and cardiovascular disease [45]. Pereira Duarte et al. found that, during the first wave of the COVID-19 pandemic, there was a significant increase in abandonment of brace therapy in AIS and a significant increase in the rate of curve progression and surgical indications among patients who voluntarily discontinued therapy [46]. This also suggests that limited or inadequate physical activity plays a negative role in the management of AIS, and the impact of reduced physical activity in adolescents on the incidence of AIS due to the consequences of the pandemic needs further study.

Physical activity is essential for adolescent growth and development, and AIS, a growth disorder, has a significant psychological and physiological impact on adolescents. There is conflicting evidence regarding the association between physical activity and AIS. To the best of our knowledge, this study is the first comprehensive and systematic assessment of the association between physical activity and AIS, suggesting that physical activity plays an important role in the prevention of AIS. Nonetheless, further in-depth studies are needed to explore the pathology and interventions in AIS.

### Limitations

This review is the first to synthesise evidence of an association between AIS and physical activity levels. However, our systematic assessment has several limitations. First, of the eight articles included in this study, five were cross-sectional studies, which did not allow a causal relationship to be established, and two were case-control studies, which did not have an adequate sample size, and there may be memory bias in tracing the physical activity levels of people with AIS through parental recall five years before and after the age of 5 years. Second, the search in this study was limited to articles published in English. Gray literature was not searched, and some of the literature that fit the search strategy was discarded because the full text was not available. This may have limited the number of studies included in our review. Third, a meta-analysis could not be conducted owing to the heterogeneity of the AIS diagnostic criteria, physical activity level measurement evaluation methods, and outcome indicators. Fourth, the overall effects of different types of exercise or physical activity were not evaluated. Lastly, the effect of nutritional status on patients with AIS should be further investigated. In the future, large-scale, prospective observational studies with longer-term follow-up are needed to further clarify the causal relationship between physical activity and AIS and to provide theoretical support for the prevention of AIS.

## Conclusions

In conclusion, there are some differences in the literature on the link between physical activity and scoliosis, depending on whether high-risk physical activity is engaged at the time and for how long, so no firm conclusions can be drawn. Studies have shown that longer periods of high-risk physical activity and lower levels of physical activity are associated with scoliosis. There is no clear consensus on whether there is a dose-relationship between levels of physical activity and idiopathic scoliosis, and large cross-sectional studies of different sports and hours of physical activity per week are needed to better determine the association between levels of physical activity and scoliosis.

### Electronic supplementary material

Below is the link to the electronic supplementary material.

**Supplementary Material 1: Appendix S1.** STROBE Statement—checklist of items that should be included in reports of observational studies

## Data Availability

All data analysed during this study are included in this published article.

## Abbreviations

AIS: Adolescent idiopathic scoliosis
BMD: Bone mineral density
WHO: World Health Organization
