# Supplementary material for: Correlation between physical activity and adolescent idiopathic scoliosis: a systematic review
Source: BMC Musculoskelet Disord. 2023 Dec 19;24:978. doi: 10.1186/s12891-023-07114-1 (PMC10729348; doi:10.1186/s12891-023-07114-1)
Supplement: Supplementary file 1 — Supplementary Material 1: Appendix S1. STROBE Statement—checklist of items that should be included in reports of observational studies [file 12891_2023_7114_MOESM1_ESM.docx]

**Appendix S1.**

STROBE Statement—checklist of items that should be included in reports of observational studies

|  | Item No | Recommendation |
| --- | --- | --- |
| Title and abstract | 1 | (a) Indicate the study’s design with a commonly used term in the title or the abstract |
|  |  | (b) Provide in the abstract an informative and balanced summary of what was done and what was found |
| Introduction | | |
| Background/rationale | 2 | Explain the scientific background and rationale for the investigation being reported |
| Objectives | 3 | State specific objectives, including any prespecified hypotheses |
| Methods | | |
| Study design | 4 | Present key elements of study design early in the paper |
| Setting | 5 | Describe the setting, locations, and relevant dates, including periods of recruitment, exposure, follow-up, and data collection |
| Participants | 6 | (a) Cohort study—Give the eligibility criteria, and the sources and methods of selection of participants. Describe methods of follow-up  Case-control study—Give the eligibility criteria, and the sources and methods of case ascertainment and control selection. Give the rationale for the choice of cases and controls  Cross-sectional study—Give the eligibility criteria, and the sources and methods of selection of participants |
|  |  | (b) Cohort study—For matched studies, give matching criteria and number of exposed and unexposed  Case-control study—For matched studies, give matching criteria and the number of controls per case |
| Variables | 7 | Clearly define all outcomes, exposures, predictors, potential confounders, and effect modifiers. Give diagnostic criteria, if applicable |
| Data sources/ measurement | 8* | For each variable of interest, give sources of data and details of methods of assessment (measurement). Describe comparability of assessment methods if there is more than one group |
| Bias | 9 | Describe any efforts to address potential sources of bias |
| Study size | 10 | Explain how the study size was arrived at |
| Quantitative variables | 11 | Explain how quantitative variables were handled in the analyses. If applicable, describe which groupings were chosen and why |
| Statistical methods | 12 | (a) Describe all statistical methods, including those used to control for confounding |
|  |  | (b) Describe any methods used to examine subgroups and interactions |
|  |  | (c) Explain how missing data were addressed |
|  |  | (d) Cohort study—If applicable, explain how loss to follow-up was addressed  Case-control study—If applicable, explain how matching of cases and controls was addressed  Cross-sectional study—If applicable, describe analytical methods taking account of sampling strategy |
|  |  | (e) Describe any sensitivity analyses |
| Results | | |
| Participants | 13* | (a) Report numbers of individuals at each stage of study—eg numbers potentially eligible, examined for eligibility, confirmed eligible, included in the study, completing follow-up, and analysed |
|  |  | (b) Give reasons for non-participation at each stage |
|  |  | (c) Consider use of a flow diagram |
| Descriptive data | 14* | (a) Give characteristics of study participants (eg demographic, clinical, social) and information on exposures and potential confounders |
|  |  | (b) Indicate number of participants with missing data for each variable of interest |
|  |  | (c) Cohort study—Summarise follow-up time (eg, average and total amount) |
| Outcome data | 15* | Cohort study—Report numbers of outcome events or summary measures over time |
|  |  | Case-control study—Report numbers in each exposure category, or summary measures of exposure |
|  |  | Cross-sectional study—Report numbers of outcome events or summary measures |
| Main results | 16 | (a) Give unadjusted estimates and, if applicable, confounder-adjusted estimates and their precision (eg, 95% confidence interval). Make clear which confounders were adjusted for and why they were included |
|  |  | (b) Report category boundaries when continuous variables were categorized |
|  |  | (c) If relevant, consider translating estimates of relative risk into absolute risk for a meaningful time period |
| Other analyses | 17 | Report other analyses done—eg analyses of subgroups and interactions, and sensitivity analyses |
| Discussion | | |
| Key results | 18 | Summarise key results with reference to study objectives |
| Limitations | 19 | Discuss limitations of the study, taking into account sources of potential bias or imprecision. Discuss both direction and magnitude of any potential bias |
| Interpretation | 20 | Give a cautious overall interpretation of results considering objectives, limitations, multiplicity of analyses, results from similar studies, and other relevant evidence |
| Generalisability | 21 | Discuss the generalisability (external validity) of the study results |
| Other information | | |
| Funding | 22 | Give the source of funding and the role of the funders for the present study and, if applicable, for the original study on which the present article is based |

Study quality was assessed using the Strengthening the Reporting of Observational Studies in Epidemiology (Checklist) for cohort, case-control, and cross-sectional studies.

**Quality assessment of included papers**

In the eight included papers, four were cross-sectional studies (and therefore considered to have a high risk of bias), one study (Tobias ,2019) was a cohort study and three studies were case-control studies (Assis, 2021, Cai, 2021, Mcmaster, 2015). Of these eight studies, only one study used accelerometers for the assessment of physical activity (Tobias ,2019) and the other seven studies used scales for the assessment of physical activity, which may have led to inaccurate results.

In Scaturro,2021, the screening for AIS in this study, although province-wide, did not specify how the sample size was selected and, in addition, the screening included only the first year of junior high school, lacking data on screening at multiple ages, which raises concerns about generalisability. Patients with AIS are in adolescence, receive many influences on their behaviour and lifestyle, and scoliosis progresses very rapidly, and screening at a single grade level could have an impact on the results

In Kenanidis, 2008, the study did not specify how the schools screened were selected, which may have led to unrepresentative results, and the lack of an objective basis for the classification of the study population may have skewed the results

In Tobias ,2019, the study did not provide a good explanation of the reasons for patient dislodgement, in addition the scoliosis data was measured using DXA, which is not the gold standard for scoliosis measurement and may have resulted in some inaccuracy in the final results.

In Golalizadeh, 2020, the study was considered to have a high risk of deflection and no radiological examination of patients with suspected scoliosis was performed, which may have influenced the results.

In Assis, 2021, the study did not perform imaging to confirm the diagnosis of suspected scoliosis, which may have resulted in inaccurate data.

In Cai, 2021 and Watanable, 2017, the studies, despite their large sample sizes, were only in a limited area and the conclusions made may not be broadly representative, and different areas and geographical settings may have different results.

In Mcmaster, 2015, the study does not explain how the sample size was determined. Also, there is no mention in the literature of some of the factors that may contribute to the risk of bias, and the data taken are scales for rating, creating too much uncertainty that can affect the results.
